# Pituitary Macroadenoma: A Comprehensive Case Study of Surgical Intervention and Postoperative Management

**DOI:** 10.7759/cureus.59387

**Published:** 2024-04-30

**Authors:** Dhruv Shah, Jayshree Sen

**Affiliations:** 1 Anaesthesia, Jawaharlal Nehru Medical College, Datta Meghe Institute of Higher Education and Research, Wardha, IND

**Keywords:** anemia, headache, multidisciplinary approach, postoperative complications, trans-nasal trans-sphenoidal surgery, pituitary macroadenoma

## Abstract

This case report presents a comprehensive analysis of a 48-year-old woman diagnosed with pituitary macroadenoma, detailing the clinical presentation, surgical intervention, and postoperative management. The patient exhibited a complex array of symptoms, including persistent headaches, insomnia, and anemia, with a history of trauma and blood transfusion. Magnetic Resonance Imaging (MRI) confirmed the presence of a large, lobulated pituitary macroadenoma, prompting a trans-nasal trans-sphenoidal endoscopic excision. The surgical procedure was successful, but postoperative complications, revealed by a CT scan, included hyperdense lesions and mixed-density collections. Incorporating antibiotics, analgesics, antacids, and anti-emetics, vigilant postoperative care addressed these complications. This case underscores the challenges and successes in managing pituitary macroadenomas, highlighting the importance of individualized care, multidisciplinary collaboration, and ongoing research for optimizing patient outcomes. The insights gained from this case contribute to the evolving understanding and refinement of strategies for addressing these complex tumors.

## Introduction

Pituitary macroadenomas are benign neoplasms originating from the anterior pituitary gland, characterized by their size exceeding 1 cm in diameter. These tumors pose a significant clinical challenge due to their potential to compress adjacent structures within the sellar and parasellar regions, leading to diverse symptoms and complications [1]. The incidence of pituitary macroadenomas is estimated to be approximately 0.1% of the population, making them relatively rare but clinically impactful [2]. The clinical presentation of pituitary macroadenomas varies widely, encompassing symptoms related to hormonal dysfunction, such as acromegaly, Cushing's disease, or hypopituitarism, as well as compressive symptoms like headaches, visual disturbances, and cranial nerve palsies [3]. Early diagnosis and appropriate management are crucial to prevent neurological deficits and improve overall patient outcomes.

The diagnostic approach to pituitary macroadenomas typically involves a combination of neuroimaging studies, including magnetic resonance imaging (MRI) with contrast and computed tomography (CT) scans, to delineate the tumor's size, location, and relationship with adjacent structures [4]. Laboratory investigations, such as hormonal assays, aid in identifying associated endocrine abnormalities. Surgical intervention remains the primary treatment modality for pituitary macroadenomas, aiming to decompress surrounding structures, alleviate symptoms, and, when necessary, address hormonal abnormalities [5]. The trans-nasal trans-sphenoidal endoscopic approach has become a widely adopted technique due to its minimally invasive nature and reduced morbidity compared to traditional approaches [6].

## Case presentation

A 48-year-old woman presented to the neurosurgery outpatient department of a tertiary care hospital, reporting persistent headaches and insomnia over the past six to seven months. During the history collection, the patient described the gradual onset and progression of the headaches, mentioning a history of a road traffic accident 2018 and a blood transfusion at the Civil Hospital. Physical examination revealed bilateral swelling around the eyes, prompting the recommendation for admission to the Neurosurgery department.

A detailed history was obtained upon admission, and blood tests and X-rays were advised. The complete blood count results indicated a haemoglobin level of 7.9 gm/dL (Reference Range: 11-15 gm/dL), indicative of anaemia. Chest X-rays showed no relevant abnormalities. Further investigation through MRI with contrast revealed a large lobulated, heterogeneously enhancing lesion in the sellar, left para sellar, suprasellar, and left temporal regions, consistent with a pituitary macroadenoma. The patient was diagnosed with pituitary macroadenoma with anaemia.

**Figure 1:**
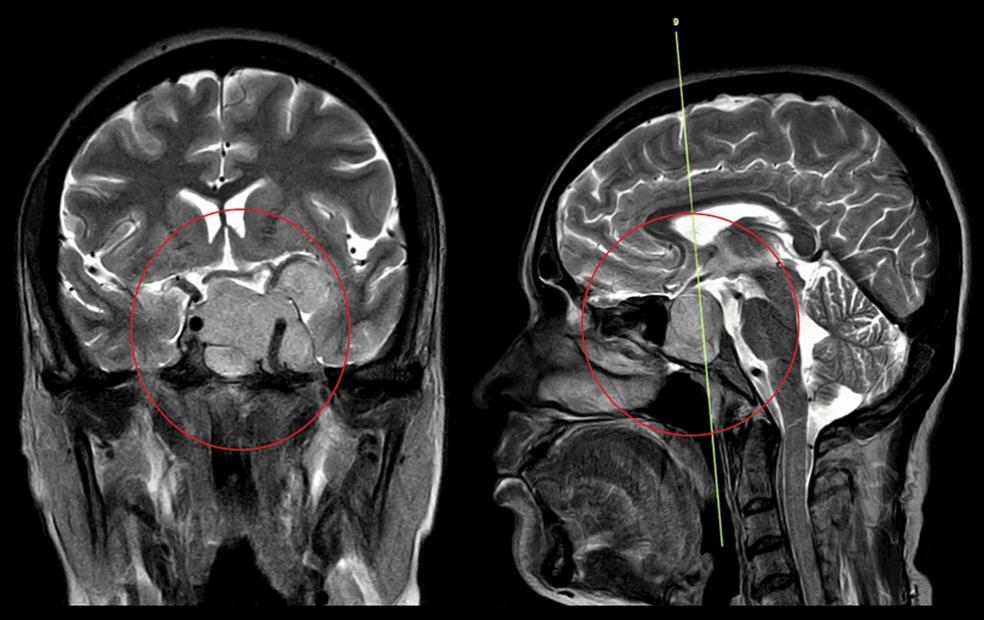
Paranasal sinuses demonstrated widening of the sella with an isodense lesion of approximately 3.3 X 1.8 cms, involving the sella and suprasellar region

A CT scan of the paranasal sinuses demonstrated widening of the sella with an isodense lesion of approximately 3.3 X 1.8 cms, involving the sella and suprasellar region. The lesion extended laterally into the left cavernous sinus and bilaterally into the sphenoid sinuses, with mucosal thickening noted in the bilateral maxillary sinuses and mild hypertrophy in the left middle and inferior turbinates (Figure 1).

The neurosurgeon explained the condition to the patient and her relative, and after counselling and obtaining consent, they planned for surgery. The patient underwent a trans-nasal trans-sphenoidal endoscopic excision of the pituitary macroadenoma under general anaesthesia. The surgical procedure involved various steps, including oral intubation, application of xylocaine + adrenaline-soaked patties, endoscopic excision of the tumour, and bony reconstruction of the post-sphenoid wall. The procedure was uneventful, and the patient received a packed red cell transfusion postoperatively.

**Figure 2:**
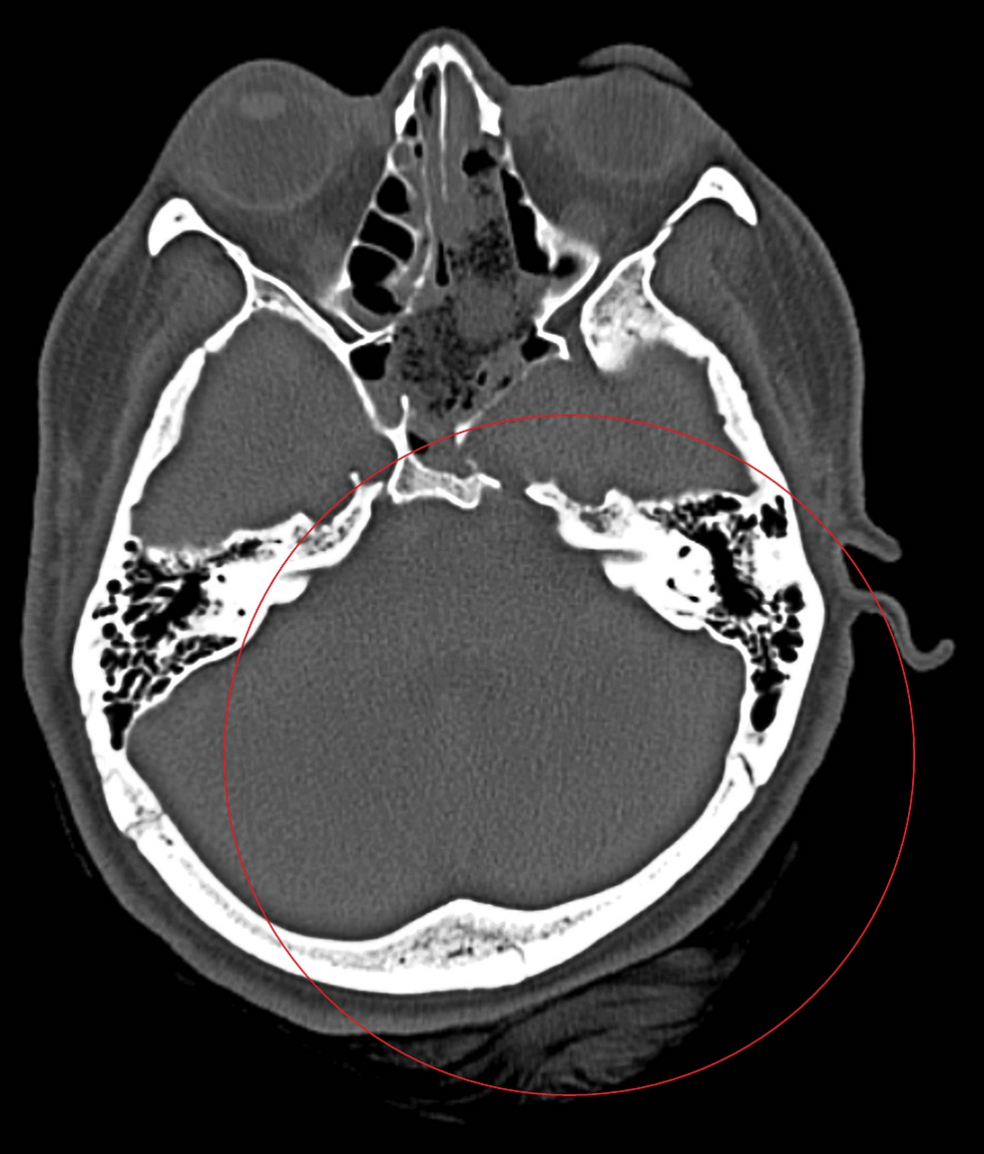
Heterogeneously hyperdense lesion in the left medial temporal region, mixed density collection in the suprasellar region

Nasal packs were removed on the second postoperative day, and a CT scan was suggested. The postoperative CT scan revealed an ill-defined, heterogeneously hyperdense lesion in the left medial temporal region, mixed density collection in the suprasellar region, and postoperative defects in the sphenoid sinus and bilateral ethmoidal sinuses (Figure 2). The patient was managed with antibiotics (ceftriaxone+sulbactam 1.5 gram), analgesics, antacids, and anti-emetics for recovery.

## Discussion

Pituitary macroadenomas present a complex clinical challenge due to their potential to cause both hormonal dysfunction and compressive symptoms. Early diagnosis and appropriate management are essential to prevent neurological deficits and improve patient outcomes [7]. This case report highlights the multidisciplinary approach required for successfully managing pituitary macroadenomas, encompassing diagnostic imaging, surgical intervention, and postoperative care. Diagnostic imaging, including MRI and CT scans, plays a crucial role in assessing pituitary macroadenomas' size, location, and extent. In this case, MRI with contrast revealed a large, lobulated lesion in the sellar, left parasellar, suprasellar, and left temporal regions, consistent with a pituitary macroadenoma [8]. The subsequent CT scan provided further details, demonstrating the lesion's involvement with adjacent structures and aiding in surgical planning.

Surgical intervention remains the cornerstone of treatment for pituitary macroadenomas, aiming to decompress surrounding structures and alleviate symptoms. The trans-nasal trans-sphenoidal endoscopic approach has emerged as a preferred technique due to its minimally invasive nature and reduced morbidity compared to traditional approaches [9]. In this case, the patient underwent successful trans-nasal trans-sphenoidal endoscopic excision of the pituitary macroadenoma, resulting in symptom relief. However, postoperative complications are not uncommon and require careful management. In this case, the postoperative CT scan revealed hyperdense lesions and mixed-density collections, necessitating antibiotic therapy and supportive care [10]. Vigilant postoperative monitoring and appropriate intervention are essential to address complications effectively and ensure optimal recovery.

The management of pituitary macroadenomas also highlights the importance of individualized care and multidisciplinary collaboration. Each patient presents with unique symptoms and comorbidities, necessitating tailored treatment strategies. Furthermore, collaboration between neurosurgeons, endocrinologists, radiologists, and other healthcare professionals is crucial for comprehensive patient care and optimal outcomes [11]. Continuous research and refinement of surgical and postoperative protocols are essential for improving patient outcomes with pituitary macroadenomas. Advances in surgical techniques, imaging modalities, and perioperative care can enhance patient safety and better long-term outcomes. Additionally, ongoing research into the molecular mechanisms underlying pituitary tumorigenesis may lead to novel therapeutic targets and personalized treatment approaches in the future [12].

## Conclusions

In conclusion, this case contributes valuable insights into the evolving landscape of pituitary macroadenoma care, emphasizing the need for continuous research and refinement of surgical and postoperative protocols to optimize outcomes for affected individuals. Overall, it reinforces the importance of personalized patient care and collaborative efforts in navigating the complexities associated with these tumours.
